# Allocation of Attentional Resources toward a Secondary Cognitive Task Leads to Compromised Ankle Proprioceptive Performance in Healthy Young Adults

**DOI:** 10.1155/2014/170304

**Published:** 2014-01-02

**Authors:** Kazuhiro Yasuda, Yuki Sato, Naoyuki Iimura, Hiroyasu Iwata

**Affiliations:** ^1^Global Robot Academia Laboratory, Green Computing Systems Research Organization, Waseda University, 27 Waseda-cho, Shinjuku-ku, Tokyo 162-0042, Japan; ^2^Graduate School of Creative Science and Engineering, Waseda University, 3-4-1 Okubo, Shinjuku-ku, Tokyo 169-8555, Japan

## Abstract

The objective of the present study was to determine whether increased attentional demands influence the assessment of ankle joint proprioceptive ability in young adults. We used a dual-task condition, in which participants performed an ankle ipsilateral position-matching task with and without a secondary serial auditory subtraction task during target angle encoding. Two experiments were performed with two different cohorts: one in which the auditory subtraction task was easy (experiment 1a) and one in which it was difficult (experiment 1b). The results showed that, compared with the single-task condition, participants had higher absolute error under dual-task conditions in experiment 1b. The reduction in position-matching accuracy with an attentionally demanding cognitive task suggests that allocation of attentional resources toward a difficult second task can lead to compromised ankle proprioceptive performance. Therefore, these findings indicate that the difficulty level of the cognitive task might be the possible critical factor that decreased accuracy of position-matching task. We conclude that increased attentional demand with difficult cognitive task does influence the assessment of ankle joint proprioceptive ability in young adults when measured using an ankle ipsilateral position-matching task.

## 1. Introduction

Ankle proprioception is critical to maintaining balance during functional activities such as standing and walking [1, 2]. Although there is general consensus on the role of visual, vestibular, and proprioceptive senses in the maintenance of upright posture [3, 4], studies have indicated that the somatosensory system is an important contributor to the feedback for postural control [5]. Previous studies have also suggested that decreased in proprioception in the lower limbs contributes significantly to instability and falls [5, 6].

In rehabilitation, proprioception should be evaluated because of its significance in motor control. Although several methods are available, the joint position-matching task is one of the most reliable tools for the assessment of proprioceptive acuity in the clinic and the laboratory [7–10]. In this test, a participant is asked to reposition a reference joint angle without observing the positioning and repositioning of the joint. Specifically, in ipsilateral position-matching tasks, where the same limb is used for reference and position matching, it is necessary to use memory in order to remember the target position [7]. Hence it is likely that some portion of any position-matching error reflects cognitive capacity.

A recent study by Goble et al. [11] reported that older adults with low working memory were prone to compromised proprioceptive encoding during an ipsilateral elbow position-matching task when a secondary cognitive task was executed concurrently. In the study, older adults with high working memory ability and those with low working memory ability, along with healthy younger adults, performed an ipsilateral elbow position-matching task with and without a secondary task (i.e., counting by 3 s) during target position encoding. The older adults with low working memory ability made significantly more elbow-repositioning errors when a secondary task was performed during target encoding, compared with both younger and older adults who had high working memory ability. The interesting conclusion of their report was that the allocation of attentional resources toward a task led to compromised sensorimotor performance because of a limitation in the resources available for concurrently coping with both tasks [11].

The current study was designed to extend previous findings and to determine whether attentional load influences ankle joint proprioceptive ability in young adults when an ipsilateral position-matching condition is adopted. We selected the ankle joints as the target joints because of the importance of ankle joints for locomotor and postural control. Previous work has shown differences during dual-task performance that included proprioception-dependent tasks, such as standing and walking [12]. In the present study, participants were instructed to perform two concurrent tasks. The primary task was an ipsilateral ankle joint position-matching task, and it was performed with or without a secondary cognitive task—a computerized auditory serial subtraction task [13]. In order to clarify the influence of the difficulty of the secondary task, we performed two experiments: one with an easy secondary task (experiment  1a) and the other with a difficult secondary task (experiment  1b). We hypothesized that increasing the difficulty of the cognitive task would result in decreased accuracy in the ankle position-matching task.

We also examined whether accurate position matching was enhanced when a position was encoded by active movement rather than passive movement. Several studies have demonstrated that participants make smaller errors when a target position is established through their own active movement than when the same target position is determined passively by the experimenter [14, 15]. The effect is thought to be the consequence of two movement-related features: an efferent copy of the motor command [16, 17] and the afferent proprioceptive information within the gamma motor system [18]. On the basis of these studies, we hypothesized that the ability to reproduce ankle position accurately is enhanced when position is encoded by active movement, compared with when it is encoded by passive movement.

## 2. Materials and Methods

### 2.1. Participants

#### 2.1.1. Experiment  1a (Easy Cognitive Task)

Sixteen young adults participated in this study. The study cohort included 8 males and 8 females, aged 25.4 ± 5.6 years, with an average body weight of 54.29 ± 5.28 kg and an average height of 162 ± 7.32 cm. The inclusion criteria were that the participants had no neurological, muscular, or hearing disorders that could influence voluntary movement and auditory sense. Participants provided written informed consent prior to participation. Waseda University's ethics committee for human research approved the procedures employed in the study. The tenets of the Declaration of Helsinki were followed.

#### 2.1.2. Experiment  1b (Difficult Cognitive Task)

A separate cohort of 16 young adults participated in experiment  1b. This cohort consisted of 8 males and 8 females, aged 26.2 ± 3.6 years, with an average body weight of 56.34 ± 6.34 kg and an average height of 161 ± 8.11 cm. The inclusion criteria were identical to those used in experiment  1a. Each participant gave written informed consent prior to participation.

### 2.2. Apparatus

We used a custom-made rotating paddle that included a rotary angle encoder (E6A2-CW3C, OMRON, Japan) to measure the joint angle and a motor (USR60-E3N, Shinsei Corporation, Japan) to move the rotating paddle. A custom-made hand switch was used to determine the target joint angle. Custom software (Visual Studio 2010, Microsoft, USA) was used to control the rotating paddle and perform real-time sampling of the joint angle. This software was also used to manipulate a computerized version of the auditory cognitive task. Headphones (HP-RX700, Victor, Japan) were used for listening during the cognitive task.

### 2.3. Tasks and Procedure

#### 2.3.1. Ipsilateral Ankle Position-Matching Task

**Figure 1:**
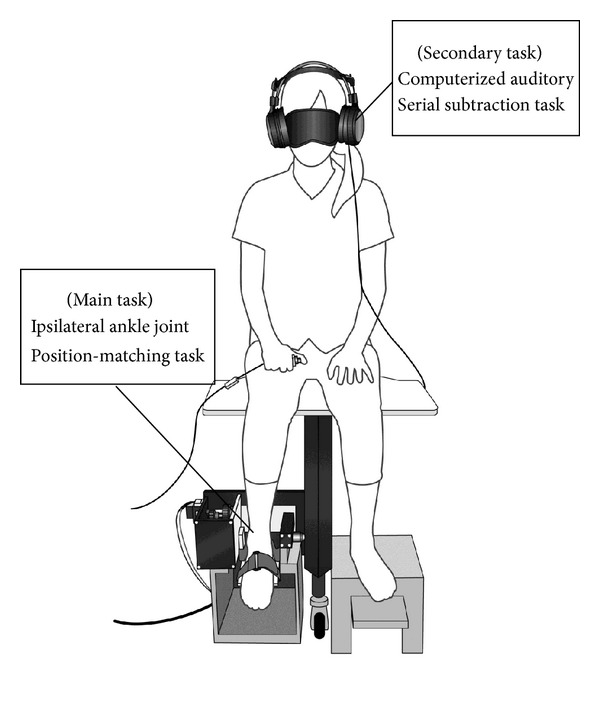
Experimental setup.

Blindfolded participants were seated with their dominant foot (right for all participants) secured with straps onto rotating paddles (Figure 1). In the target-encoding phase, the participants plantarflexed the ankle joint to 20° or 25° from the 0° (neutral) position using either active movement or operation of the rotating paddle (passive movement). This target position was maintained for 12 s, allowing the participants to encode it into their memories. After the target-encoding phase, the ankle was returned to the start position, and then the paddle automatically moved the ankle toward the target angle. The participants were asked to press a hand switch when they felt that their ankle had reached the target angular position. Participants performed the position-matching task 9 times, and the target joint angle was randomly assigned.

#### 2.3.2. Cognitive Task (Computerized Version of the Auditory Serial Subtraction Task)

The cognitive task was a computerized version of the auditory serial subtraction task (ASST), in which participants were instructed to continuously subtract a selected number from a randomly selected two-digit number [13, 19]. Participants performed the subtraction task during the 12 s position-encoding phase. The initial number and the pitch of ASST were produced through the headphones, while participants had to provide an answer for ASST verbally. ASST was selected because (a) processing of the task was based on mental arithmetic and (b) it allowed the investigation of different levels of cognitive difficulty and manipulation of the pitch with a programming algorithm.

In experiment  1a, the participants were asked to continuously subtract 3 from the randomly selected two-digit number for 3 s during the 12 s position-encoding phase (i.e., 4 subtraction tasks during 12 s). In experiment  1b, the participants were instructed to continuously subtract 7 from a randomly selected two-digit number for 1 s during the 12 s position-encoding phase (i.e., 12 subtraction tasks during 12 s).

#### 2.3.3. Procedure

**Figure 2:**
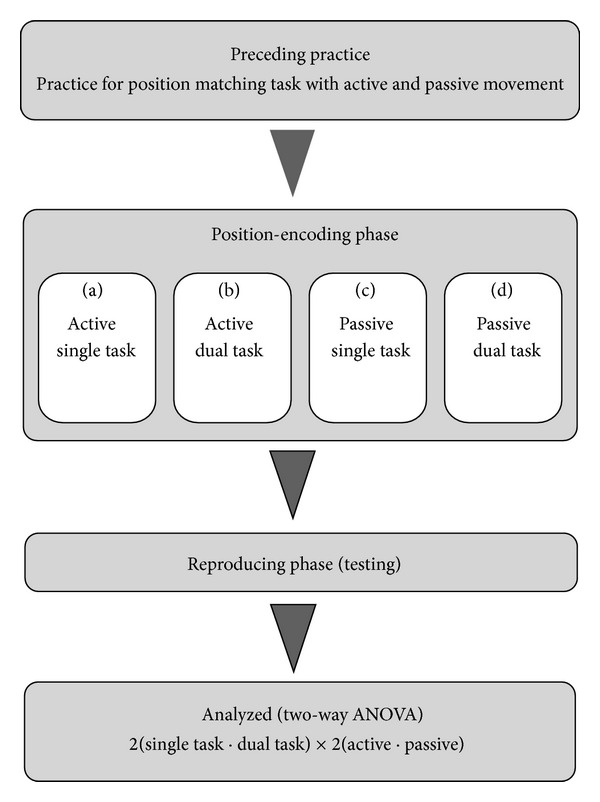
Experimental procedure flow diagram.

The entire procedure (Figure 2) was carried out in an experiment  room. At the beginning of the experiment, the participants practiced an ipsilateral position-matching task, in which they tried as accurately as possible to detect a predefined target angle. Two minutes after the end of the initial practice session, the participants moved on to the experimental session. Participants were subjected to 2 (single task, dual task) × 2 (active, passive) experimental conditions for the ankle position-matching task. The conditions were defined as follows: (a) AS, active × single task; (b) AD, active × dual task; (c) PS, passive × single task; (d) PD, passive × dual task. The order of these conditions was counterbalanced among the participants.

### 2.4. Outcome Measurements

#### 2.4.1. Position-Matching Errors

Three common error scores were used to measure movement accuracy [18]: AE, absolute error; CE, constant error; VE, variable error. AE is the measurement of the magnitude of the error regardless of the direction. CE is the measurement of response bias in relation to a target. VE measures the consistency of the movement.

Each mean AE of joint reproduction was calculated using the following formula:

(1) $$\begin{array}{c} \text{AE}=\frac{\sum \left|X-C\right|}{K}. \end{array}$$

In this formula, AE represents the sum of the error for each trial divided by the number of trials in the block. The variable *X* represents the raw score, *C* represents the criterion score desired, and *K* represents the number of trials. The sign of the value of *X* is to be ignored when calculating AE.

CE was calculated using the following formula:

(2) $$\begin{array}{c} \text{CE}=\frac{\sum \left(X-C\right)}{K}. \end{array}$$

This formula is similar to AE except that the relative sign of each score is considered. VE was calculated using the following formula:

(3) $$\begin{array}{c} \text{VE}=\sqrt{\frac{{\sum \left(X-C\right)}^{2}}{K}-{\left(\text{CE}\right)}^{2}.} \end{array}$$

In this formula, VE is calculated by taking the square root of the sum of the squared difference between each individual error score and the CE score for that block divided by the number of trials in the block.

#### 2.4.2. Percentage of Correct Answers to the Cognitive Task

Each time a participant gave an answer during the subtraction task, it was determined to be correct or incorrect. These data were used to calculate the percentage of correct answers.

### 2.5. Statistical Analysis

For AE, CE, and VE, a separate statistical analysis was performed. A two-way (attention, movement patterns) analysis of variance (ANOVA) with repeated measures on both factors was performed on each dependent variable. A *P* value of <0.05 was considered statistically significant. A post hoc comparison using Bonferroni's multiple comparison was performed to determine which comparisons were different.

## 3. Results

### 3.1. Experiment  1a

#### 3.1.1. Position-Matching Errors

**Figure 3:**
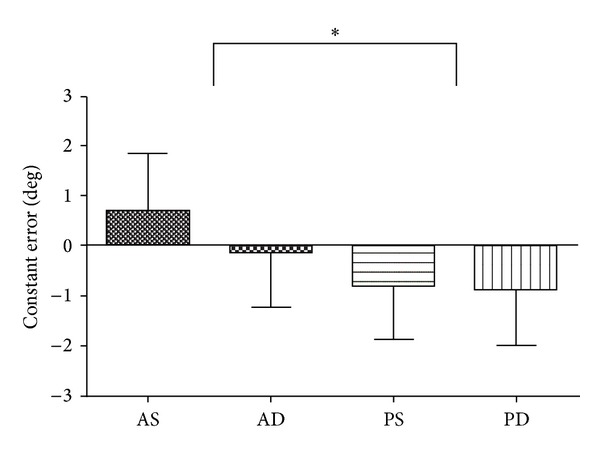
Mean ± SD constant errors for each condition in experiment  1a. AS: active/single task, AD: active/dual task, PS: passive/single task, PD: passive/dual task.

The means and standard deviations in the position-matching errors AE, CE, and VE are shown in Table 1. For CE, a two-way ANOVA showed that the main effect, the movement pattern, was significant (*F*(1,60) = 16.14,  *P* < 0.01) (Table 2, Figure 3), which indicated that participants made smaller errors when matching a reference position that was established through passive movement rather than active movement. No other main effects or interactions were significant for any of the measurements.

#### 3.1.2. Percentage of Correct Answers to the Cognitive Task

The average percentage of correct answers to the cognitive task in the AD condition was 98.4% ± 3.35% and was 97.9% ± 4.68% in the PD condition.

### 3.2. Experiment  1b (Difficult Cognitive Task)

#### 3.2.1. Position-Matching Errors

**Figure 4:**
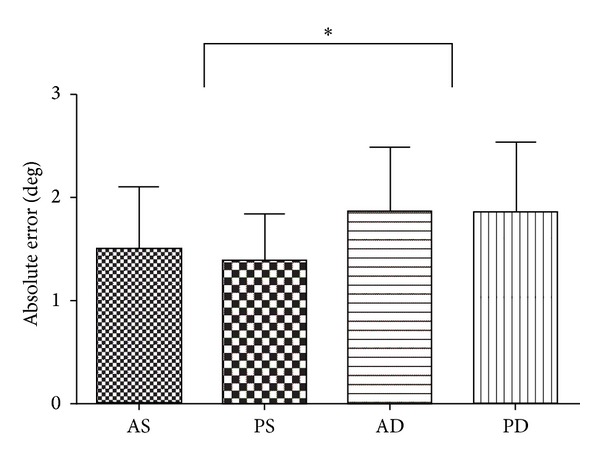
Mean ± SD absolute errors for each condition in experiment  1b AS: active/single task, PS: passive/single task, AD: active/dual task, PD: passive/dual task.

The means and standard deviations of each position-matching error are shown in Table 3. For AE, a 2-way ANOVA showed that the main effect, attention, was significant [*F*(1,60) = 6.80, *P* < 0.05] (Table 4, Figure 4), which indicated that participants made significantly greater errors in the dual-task conditions than in the single-task conditions. No other main effects or interactions were significant for any of the measurements.

#### 3.2.2. Percentage of Correct Answers to the Cognitive Task

The average percentage of correct answers to the cognitive task in the AD condition was 56.1% ± 18.19%; in the PD condition, it was 57.9% ± 18.76%.

## 4. Discussion

In this study, young adults performed an ipsilateral ankle position-matching task in single- and dual-task conditions. The goal of these experiments was to extend previous findings and to determine whether attentional load influences ankle joint proprioceptive ability in young adults when an ipsilateral position-matching paradigm is adopted. The results showed that participants made significantly more ankle position-matching errors under difficult dual-task conditions than under single-task conditions. These findings support previous findings that allocation of attentional resources toward a secondary task can lead to compromised sensorimotor performance, because of a limitation in the available resources for dealing with two tasks concurrently. Furthermore, we found that the difficulty level of the cognitive task might be the possible critical factor that decreased the accuracy of the position-matching task under dual-task conditions.

To our knowledge, this is the first study to demonstrate that increased attentional demands influence the acuity of the ipsilateral ankle position-matching task in young adults. This result suggests that allocation of limited attentional resources during dual-task conditions interferes with the encoding of ankle position. The reduction in ipsilateral ankle position-matching accuracy seen under the difficult dual-task condition suggests that encoding proprioceptive target information shares a common neural substrate with the ability to allocate memory to encode and perform an arithmetic task. Previous research has shown that older adults with low working memory ability made significantly more elbow-repositioning errors when secondary tasks were present during target encoding than both younger and older adults with high working memory ability [11]. In the present study, we selected the ankle joints as the target joints, and in order to clarify the effects of the difficulty of the secondary task, we performed two experiments with two different cohorts: one in which the auditory subtraction task was easy (experiment  1a) and one in which it was difficult (experiment  1b). Our results allow for a streamlined interpretation of the finding that the difficulty of the cognitive task influenced performance in the ipsilateral ankle position-matching task, implying that there is a role for attentional resource in the assessment of ankle proprioception with an ipsilateral position-matching task.

In the present study, although position-matching accuracy (i.e., AE) was not enhanced by active movement during the position-encoding phase of experiment  1a, the results in CE showed that the participants produced undershoot when matching a target position established through passive movement than when matching the same target position encoded actively. This result indicates that the participants tended to underestimate the target position when it was encoded using the passive movement of a rotatory paddle. Some previous studies have demonstrated that CE is not dependent on the mode of target presentation [20, 21], whereas other studies have demonstrated that passive target presentation results in overshooting [22, 23]. These inconsistent findings suggest that the undershooting of passively generated target positions is task dependent. Furthermore, as this tendency was not replicated in experiment  1b, further studies are necessary to clarify the exact nature of this effect.

Given that most proprioceptive assessments conducted in clinical environments use position-matching tasks administered by therapists, clinicians should note that some portion of the position-matching error reflects cognitive or memory capacity, rather than proprioception itself, when ipsilateral position matching is used in individuals who are prone to having memory issues, such as older people or stroke patients. Furthermore, a previous study has reported differences in dual-task performance that included proprioception-dependent tasks, such as standing and walking [12]. For instance, Harley et al. reported that increased attentional demands during an obstacle-crossing task led to a decrease in obstacle clearance or increased variability in older adults [24]. They predict that, as attention demands increased further, older individuals would show greater reductions in obstacle-crossing performance. When clinical training contains the memory of a movement (i.e., ascending the stairs or stepping over obstacles), cognitive aspects should be taken into account during the assessment of training performance.

There is a methodological issue that limits the conclusions to be drawn from this study. Because the experiments were conducted with relatively young participants, other cohorts where these results could potentially be more relevant (i.e., stroke patients or elderly people) should be included in future studies to exclude the potential for bias occurring in this group of participants. Another limitation of the study is that different cohorts were chosen for the easy (1a) and difficult (1b) tasks in the present study. The reason for the choice of two different cohorts was to avoid a learning effect (i.e., by allowing the participants to become familiar with the dual task procedure). In our pilot study, it became apparent that if someone experiences a difficult cognitive task, they would be able to perform an easier cognitive task without requiring a cognitive load. Thus, we conducted this research with two different cohorts. However, there might be a risk that the difference found was a group effect, rather than an effect of the cognitive task complexity. This is an important issue that warrants additional research (e.g., comparing randomised easy and difficult tasks within the same cohort). In future work, it would also be of interest to address this possibility using a more extensive battery of cognitive tests and to compare the results to standardized norms. Our results raise the question whether other cognitive factors might also influence performance on tests of ankle proprioceptive performance.

## 5. Conclusion

The present study showed that performing a secondary cognitive task resulted in decreased ipsilateral ankle position-matching performance relative to single-task conditions in young healthy participants. This tendency was observed only for the cohort that performed a difficult cognitive task. Therefore, the difficulty level of the cognitive task might be the possible critical factor that decreased accuracy in the position-matching task under dual-task conditions. These results indicate that allocation of attentional resources toward a difficult cognitive task can lead to compromised sensorimotor performance, because of a limitation in resources available for concurrently coping with both tasks. Further studies that include a larger sample and greater diversity of individuals are necessary to validate our conclusions and findings in a clinical setting.

## Figures and Tables

**Table 1:** Average joint position-matching errors in experiment 1a (mean ± standard deviation).

	Active/single	Active/dual	Passive/single	Passive/dual
AE	1.73 ± 0.52	1.44 ± 0.46	1.54 ± 0.72	1.63 ± 0.61
CE	0.67 ± 1.16	−0.14 ± 1.09	−0.81 ± 1.06	−0.87 ± 1.12
VE	1.68 ± 0.75	1.49 ± 0.48	1.45 ± 0.47	1.38 ± 0.53

AE: absolute error (degrees); CE: constant error (degrees); VE: variable error (degrees).

**Table 2:** Two-way ANOVA results of the joint position-matching error parameters in experiment 1a. *P* values derived from ANOVA for the main effects of attention and movement. Interaction between attention and movement.

Joint position-matching errors (*n* = 16)		
	F	*P*
AE		
Attention (A)	0.43	0.51
Movement (B)	0.00	0.97
A × B	1.73	0.19

CE		
Attention (A)	2.53	0.11
Movement (B)	16.14	0.00**
A × B	1.80	0.18

VE		
Attention (A)	0.82	0.37
Movement (B)	1.38	0.24
A × B	0.19	0.65

AE: absolute error; CE: constant error; VE: variable error. **P < 0.01.

**Table 3:** Average joint position-matching errors in experiment 1b (mean ± standard deviation).

	Active/single	Active/dual	Passive/single	Passive/dual
AE	1.56 ± 0.59	1.88 ± 0.65	1.43 ± 0.46	1.90 ± 0.66
CE	−0.15 ± 0.78	−0.19 ± 1.53	−0.37 ± 0.86	−0.33 ± 1.39
VE	1.72 ± 0.54	1.91 ± 0.61	1.44 ± 0.37	1.70 ± 0.43

AE: absolute error (degrees); CE: constant error (degrees); VE: variable error (degrees).

**Table 4:** Two-way ANOVA results of the joint position-matching error parameters in experiment 1b. *P* values derived from ANOVA for the main effects of attention and movement. Interaction between attention and movement.

Joint position-matching errors (*n* = 16)		
	F	*P*
AE		
Attention (A)	6.80	0.01 *
Movement (B)	0.14	0.71
A × B	0.27	0.60

CE		
Attention (A)	0.00	1.00
Movement (B)	0.38	0.53
A × B	0.02	0.89

VE		
Attention (A)	3.27	0.07
Movement (B)	3.99	0.05
A × B	0.10	0.75

AE: absolute error; CE: constant error; VE: variable error. *P < 0.05.
